# Supplementary material for: Reassessing taxonomy and virulence in the Fusobacterium nucleatum group—rebuttal of Fusobacterium animalis clades “Fna C1” and “Fna C2,” genome announcement for Fusobacterium watanabei, and description of Fusobacterium paranimalis sp. nov
Source: mBio. 2025 Jul 31;16(9):e00941-25. doi: 10.1128/mbio.00941-25 (PMC12421844; doi:10.1128/mbio.00941-25)
Supplement: File S1 — Generation and comparisons of MALDI-ToF reference spectra. [file mbio.00941-25-s0001.docx]

Supplementary File 1

# **Generation of MALDI-ToF reference spectra for *F. watanabei* and *F. paranimalis***

The two type strains were cultured on acumedia® blood agar plates (Neogen) and duplicated full formic acid extraction was done thereafter. After creation of 30 spectra per strain, with concomitant Bacterial Test Standard control spectrum generation, adjustment and smoothing of the baseline as well as removal of poor quality spectra was done in FlexAnalysis Version 3.4 (build79)(Bruker). Reference spectra were then created using BioTyper Explorer (Bruker).

We then proceeded with comparing the reference spectra of *F. paranimalis* and *F. watanabei* to reference spectra of other *Fusobacterium nucleatum* group members available in the standard clinical Bruker MALDI Biotyper database (MBT Compass Library 2023 (12438MSP)), with the aim of identifying distinct spectral peaks for each species and to assess whether the novel spectra were distinguishable to spectra representing other species.

The Bruker software can produce dendrograms comparing MALDI-ToF spectra and in a neighbour-joining fashion group spectra together based on spectral similarities. The reference spectra of both *F. paranimalis* and *F. watanabei* grouped together with the other members of the *F. nucleatum* group, but *F. paranimalis* was considerably closer to *F. animalis* spectra compared to *F. watanabei* (Suppl. Fig. 3)*.*

For *F. paranimalis*, the closest *F. nucleatum* group reference spectra all received <1.8 similarity score, reflecting good distinction between *F. paranimalis* and other closely related species. When compared to closest hits, the majority of *F. paranimalis* reference spectrum peaks were unique when compared to *F. watanabei, F. vincentii* and *F. animalis* (red peaks in upper parts of spectral comparison plots, Suppl.Fig.4).

The *F. watanabei* spectrum was even more distinct, with a similarity score <1,6 against reference spectra of other *F. nucleatum* group species . Compared to the closest two species, *F. paranimalis* and *F. canifelinum*, yet again, most peaks were distinct to *F. watanabei* (red peaks in upper parts of spectral comparison plots, Suppl.Fig.5).

The two reference spectra for *F. paranimalis* and *F. watanabei* are enclosed as a zipped file (Supplementary File 2). We will also send the *F. paranimalis* spectrum/culture to Bruker for inclusion of this species into the Bruker MALDI Biotyper database in the future.


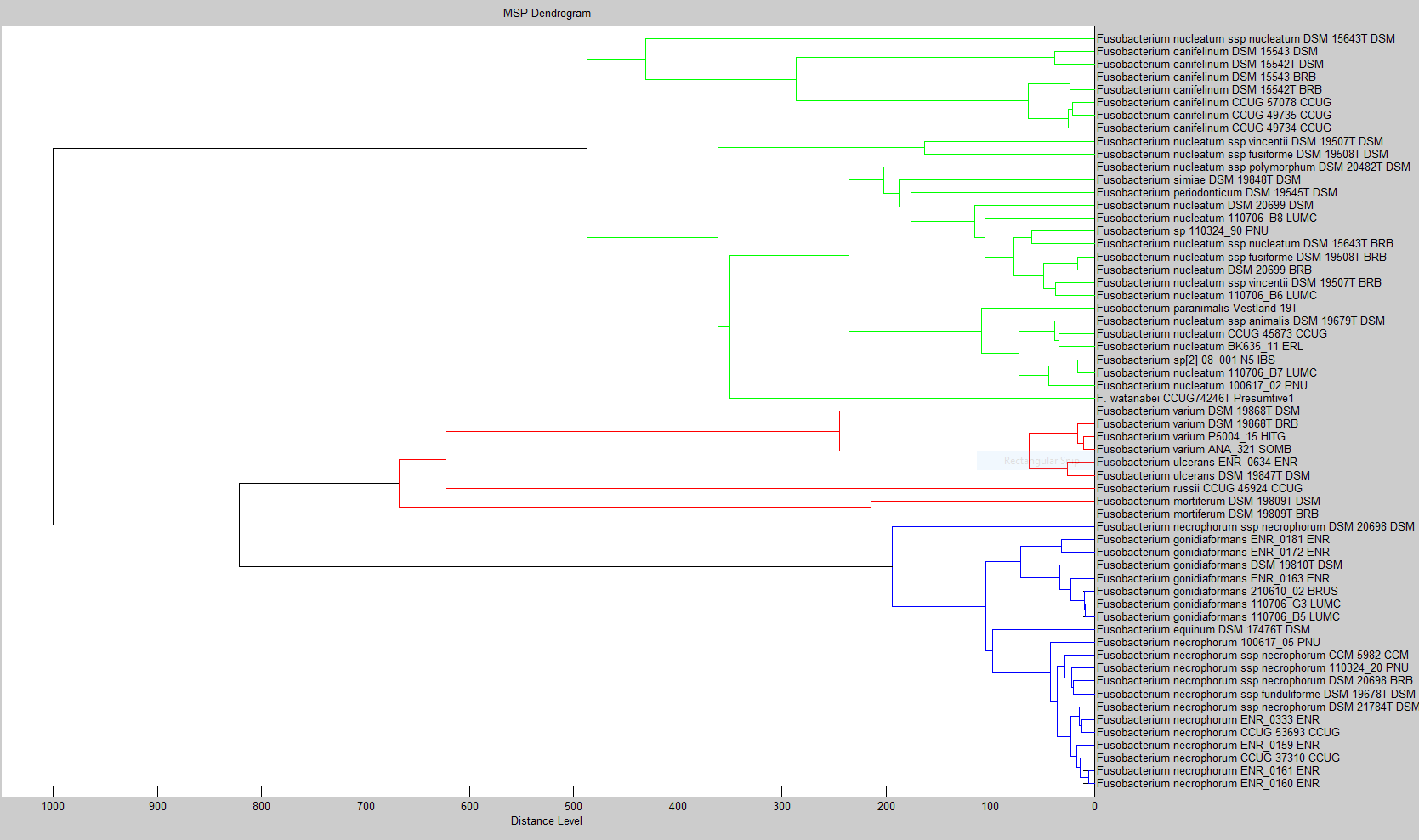


Suppl. figure 3: Dendrogram output comparing similarity between all available *Fusobacterium* spp. spectra with *F. watanabei* and *F. paranimalis* reference spectra included.


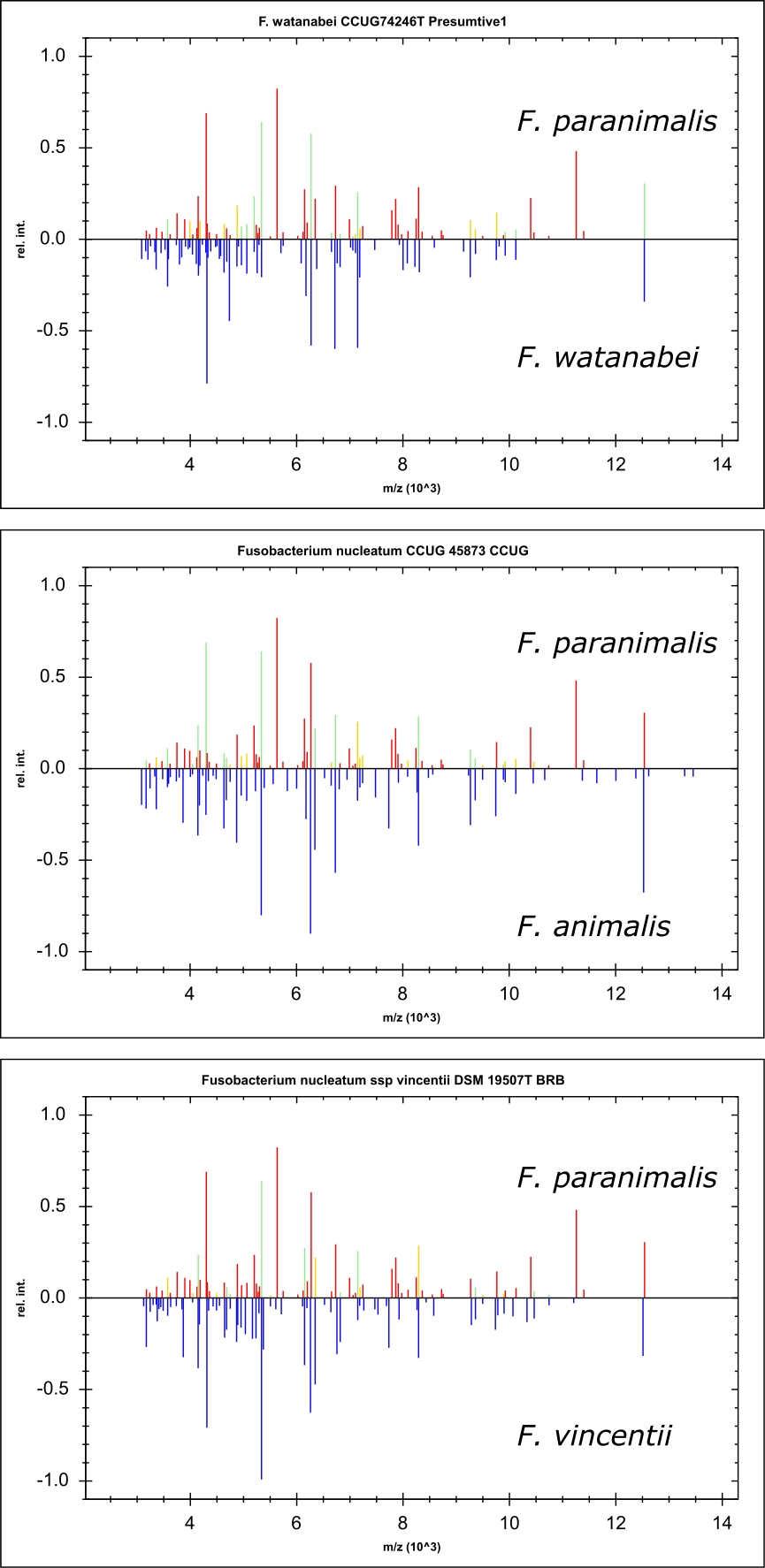


Suppl. Fig. 4: Reference spectrum of *F. paranimalis* compared with *F. watanabei* (upper), *F. animalis* (mid) and *F. vincentii* (lower), with the *F. paranimalis* reference spectrum on top. Color indications indicate perfect peak match (green), poor peak match (yellow) and no peak match (red). Blue peaks indicate comparator spectrum.


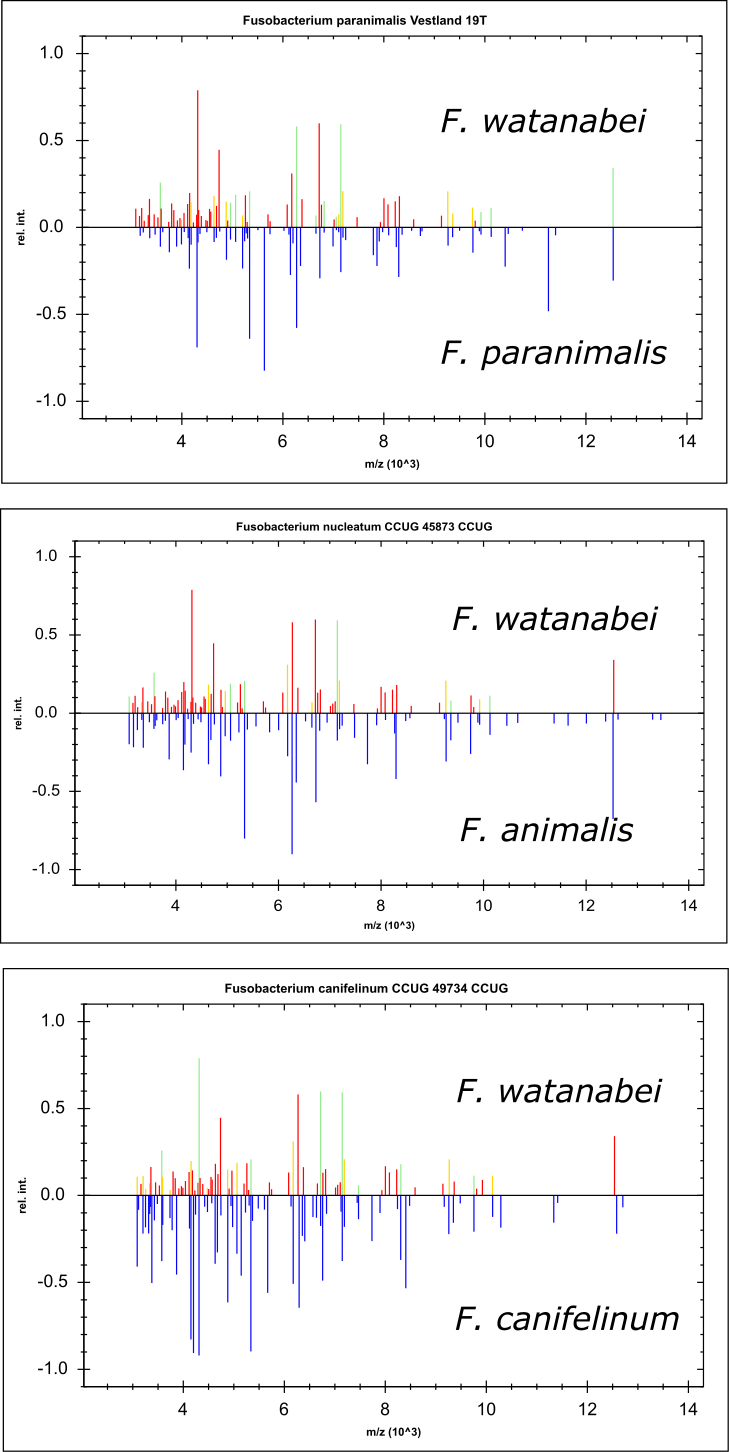


Suppl. Fig. 5: Reference spectrum of *F. watanabei* compared with *F. paranimalis* (upper), *F. animalis* (mid) and *F. canifelinum* (lower), with the *F. watanabei* reference spectrum on top. Color indications indicate perfect peak match (green), poor peak match (yellow) and no peak match (red). Blue peaks indicate comparator spectrum. Bruker reports the top ten hits, of which none were *F. animalis*.
