# Supplementary material for: Reassessing taxonomy and virulence in the Fusobacterium nucleatum group—rebuttal of Fusobacterium animalis clades “Fna C1” and “Fna C2,” genome announcement for Fusobacterium watanabei, and description of Fusobacterium paranimalis sp. nov
Source: mBio. 2025 Jul 31;16(9):e00941-25. doi: 10.1128/mbio.00941-25 (PMC12421844; doi:10.1128/mbio.00941-25)
Supplement: Fig. S1 — Splits tree of Fusobacterium spp. [file mbio.00941-25-s0002.pdf]

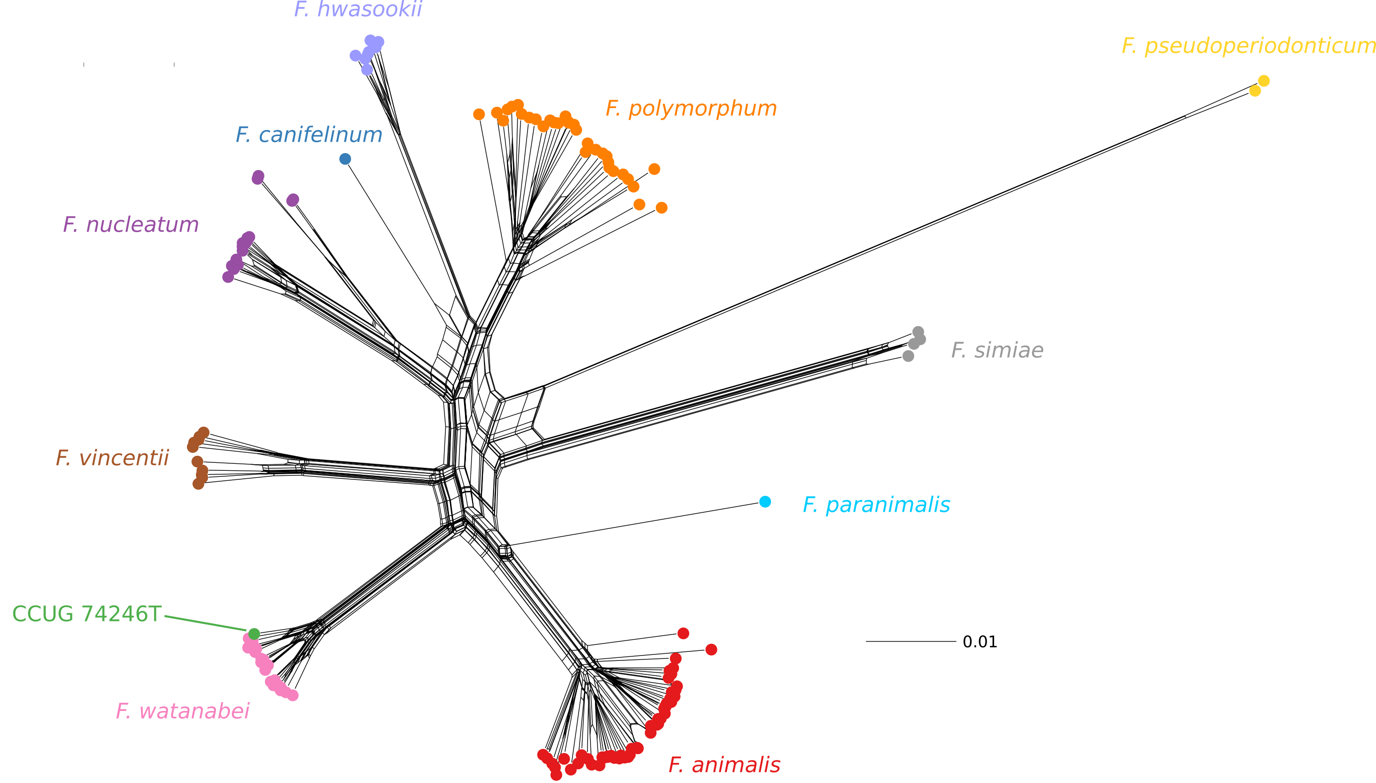

Supplementary Figure S1. Neighbor-net split network of fusobacterial core genomes. Each sequence is shown as a dot, color-coded by species. Within the *F. watanabei* cluster, the placement of the type strain CCUG 74246T is shown.
