# Supplementary material for: Reassessing taxonomy and virulence in the Fusobacterium nucleatum group—rebuttal of Fusobacterium animalis clades “Fna C1” and “Fna C2,” genome announcement for Fusobacterium watanabei, and description of Fusobacterium paranimalis sp. nov
Source: mBio. 2025 Jul 31;16(9):e00941-25. doi: 10.1128/mbio.00941-25 (PMC12421844; doi:10.1128/mbio.00941-25)
Supplement: Fig. S2 — Light microscopy of F. nucleatum, F. paranimalis sp. nov., and F. watanabei. [file mbio.00941-25-s0003.pdf]

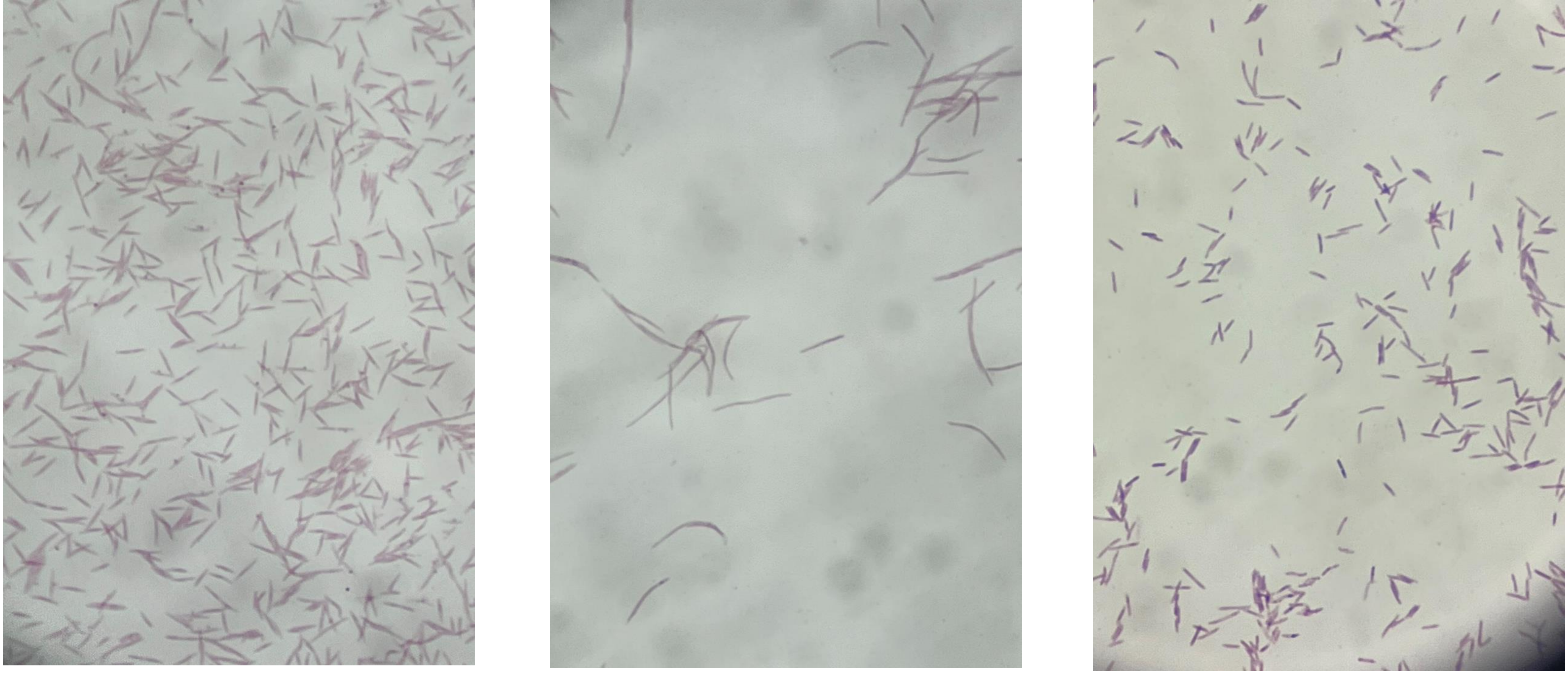

Fusobacterium nucleatum
ATCC 23726

Fusobacterium paranimalis sp. nov.
Vestland19

Fusobacterium watanabei
CCUG74246

Supplementary figure S2. Light microscope morphology of *F. nucleatum, F. paranimalis,* and *F. watanabei.*
